# Autoimmune Hepatitis Presenting With Severe Pruritus in a 27-Year-Old Male

**DOI:** 10.7759/cureus.99742

**Published:** 2025-12-20

**Authors:** Nazmus Sakib, Mymuna Binte Mukarrom

**Affiliations:** 1 Gastroenterology and Hepatology, Square Hospitals Limited, Dhaka, BGD; 2 Medicine, Shaheed Suhrawardy Medical College and Hospital, Dhaka, BGD

**Keywords:** atypical hepatic presentation, autoantibodies, autoimmune hepatitis, azathioprine, liver inflammation, prednisolone therapy, pruritus, transaminase elevation

## Abstract

Autoimmune hepatitis (AIH) is a chronic immune-mediated liver disorder characterized by progressive hepatocellular inflammation and elevated autoantibodies. Although most patients present with jaundice, fatigue, or nonspecific hepatic symptoms, pruritus as the primary complaint is uncommon, especially in the absence of overt cholestasis. We report a case of a 27-year-old male presenting with severe generalized pruritus who was ultimately diagnosed with AIH. Laboratory evaluation revealed a hepatocellular injury pattern, strongly positive autoimmune serology, and elevated IgG levels, consistent with AIH. High-dose prednisolone therapy led to marked symptomatic improvement and rapid biochemical normalization. This case highlights pruritus as an uncommon but important presentation of AIH and underscores the need to consider AIH in patients with persistent, unexplained pruritus.

## Introduction

Autoimmune hepatitis (AIH) is a chronic inflammatory liver disease characterized by circulating autoantibodies, elevated serum IgG, and interface hepatitis on histology [1]. The condition exhibits substantial clinical heterogeneity, with presentations ranging from incidental elevations in aminotransferases to acute severe hepatitis or even acute liver failure, making early diagnosis challenging [2]. Although classic symptoms include fatigue, jaundice, and abdominal discomfort, atypical manifestations may occur. Pruritus, a hallmark symptom of cholestatic liver diseases, is not commonly associated with AIH and may therefore lead clinicians to consider alternative hepatobiliary or dermatologic diagnoses, contributing to diagnostic delays [3].

Timely recognition and prompt initiation of immunosuppressive therapy are essential, as untreated AIH may progress rapidly to cirrhosis, portal hypertension, and hepatic decompensation [1]. In contrast, corticosteroid-based therapy is associated with excellent biochemical response rates and favorable long-term survival when initiated early in the disease course [4]. Awareness of unusual presentations, particularly in young patients presenting with refractory pruritus and a hepatocellular enzyme pattern, is crucial to prevent delays in diagnosis and mitigate disease progression.

## Case presentation

Patient history

A 27-year-old previously healthy male presented with a four-week history of severe, generalized pruritus, most prominent at night and significantly disrupting sleep. The itching was diffuse and unresponsive to antihistamines, emollients, or a short course of low-dose systemic corticosteroids prescribed during a prior dermatology consultation. He did not report jaundice, dark urine, pale stools, abdominal pain, fever, weight loss, recent medication or supplement use, alcohol intake, or recreational drug use. There was no personal or family history of autoimmune or chronic liver disease. The family history was specifically reviewed for atopic conditions, autoimmune disorders, and cholestatic liver diseases, such as primary biliary cholangitis (PBC) and primary sclerosing cholangitis (PSC), given their association with generalized pruritus; none were reported.

This information was obtained during the initial assessment to evaluate systemic causes of persistent pruritus. Because the patient had severe generalized pruritus without primary skin lesions, a systemic cause, including possible hepatic dysfunction, was considered early in the evaluation. He presented to our clinic after four weeks of persistent symptoms, at which point baseline laboratory investigations were obtained to assess for systemic or hepatic causes of pruritus.

Examination findings

Physical examination revealed multiple linear excoriation marks across the trunk and limbs. Mild scleral icterus was noted, despite the patient not having noticed any yellowing of the eyes. This finding was observed even with only mildly elevated serum bilirubin levels, as subtle scleral icterus can be clinically appreciable early in inflammatory hepatocellular liver disease before marked hyperbilirubinemia develops. No peripheral stigmata of chronic liver disease were present. Abdominal examination demonstrated mild, nontender hepatomegaly without splenomegaly or ascites. Cardiovascular, respiratory, and neurological examinations were unremarkable.

Investigations

Baseline laboratory evaluation at the initial visit demonstrated a marked hepatocellular pattern of injury, with alanine aminotransferase (ALT) at 612 U/L and aspartate aminotransferase (AST) at 545 U/L. Alkaline phosphatase was mildly elevated at 211 U/L, and gamma-glutamyl transferase (GGT) was markedly elevated at 521 U/L. Total bilirubin was 1.4 mg/dL, and serum albumin was slightly reduced at 3.4 g/dL. Coagulation parameters were preserved, with an international normalized ratio (INR) of 0.90.

Autoimmune serology showed strongly positive antinuclear antibody (ANA >4000 AU/mL, speckled pattern) and anti-smooth muscle antibody (ASMA) at 1:80. Anti-liver-kidney microsomal antibody, anti-double-stranded DNA antibody, and anticardiolipin antibodies were all negative. Serum IgG was elevated at 1700 mg/dL.

Follow-up biochemistry after initiation of corticosteroid therapy demonstrated progressive improvement in liver enzymes. At two weeks, ALT decreased to 283 U/L, AST to 146 U/L, and GGT to 245 U/L. At four weeks, ALT was 158 U/L, AST 52 U/L, and GGT 128 U/L, with bilirubin reduced to 0.6 mg/dL and albumin improved to 3.8 g/dL. By six weeks, ALT further declined to 65 U/L, AST to 45 U/L, and GGT to 70 U/L, with bilirubin remaining at 0.6 mg/dL. INR remained stable at 1.1 at two and four weeks. Table 1 summarizes the patient’s serial liver biochemistry from baseline to six weeks.

**Table 1:** Serial liver biochemistry from baseline to six weeks Reference ranges are based on standard adult laboratory values. Albumin and INR were not repeated at all follow-up time points due to early clinical improvement and marked biochemical response to immunosuppressive therapy. ALP, alkaline phosphatase; ALT, alanine aminotransferase; AST, aspartate aminotransferase; GGT, gamma-glutamyl transferase; INR, international normalized ratio

Parameter	Reference range	Baseline	Two weeks	Four weeks	Six weeks
ALT (U/L)	7-56	612	283	158	65
AST (U/L)	10-40	545	146	52	45
ALP (U/L)	44-147	211	121	66	68
GGT (U/L)	8-61	521	245	128	70
Total bilirubin (mg/dL)	0.1-1.2	1.4	1.1	0.6	0.6
Albumin (g/dL)	3.5-5.0	3.4	-	3.8	-
INR	0.8-1.2	0.9	1.1	1.1	-

**Figure 1:**
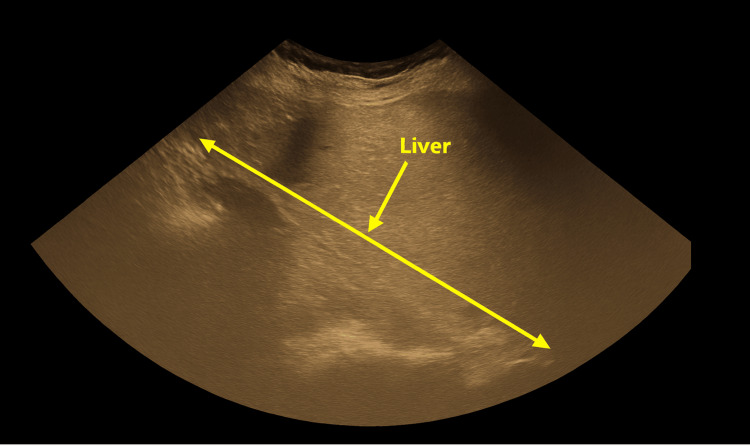
Ultrasound image demonstrating mild hepatomegaly with preserved parenchymal echotexture The long arrow indicates the craniocaudal liver span, and the short arrow marks the hepatic parenchyma, which appears homogeneous without focal lesions. Formal ultrasonography did not reveal any features of biliary obstruction.

Ultrasound imaging demonstrated mild hepatomegaly with preserved parenchymal echotexture and no intrahepatic or extrahepatic biliary duct dilation. Figure 1 illustrates the ultrasound findings.

Management and follow-up

High-dose oral prednisolone (60 mg/day) was initiated at the initial hepatology assessment immediately after baseline investigations supported the clinical suspicion of AIH. This led to rapid symptomatic improvement and a significant reduction in liver enzymes after two weeks. The pruritus did not improve spontaneously and showed marked relief only following the initiation of corticosteroid therapy.

Azathioprine was introduced at a fixed starting dose of 50 mg/day as a steroid-sparing agent after confirming normal thiopurine methyltransferase (TPMT) activity, with plans for further titration based on tolerance and biochemical response. The patient was advised to continue prednisolone with a planned gradual taper according to biochemical response and clinical improvement, and follow-up was scheduled at two weeks.

At the four-week follow-up, the patient reported marked but incomplete improvement of pruritus, and liver enzymes showed a sharp decline, indicating an early favorable response. By six weeks, pruritus had further improved, limited only to the thighs, and liver enzymes were nearly within normal limits, demonstrating substantial ongoing biochemical recovery.

## Discussion

AIH is a chronic, immune-mediated inflammatory liver disease with a wide spectrum of clinical presentations, ranging from asymptomatic transaminase elevation to acute severe hepatitis or liver failure. The disease is characterized by hepatocellular inflammation, hypergammaglobulinemia, and the presence of autoantibodies such as ANA and ASMA [1]. Despite advancements in diagnostic criteria, AIH remains a diagnostic challenge due to its heterogeneous manifestations and overlap with other liver and autoimmune conditions.

The classical presentation of AIH includes jaundice, fatigue, malaise, anorexia, and abdominal discomfort. However, up to one-third of patients may initially present with nonspecific or atypical symptoms, contributing to diagnostic delay [2]. Among these atypical features, isolated pruritus, as seen in this case, is particularly uncommon. Large clinical series of AIH describe pruritus infrequently and usually in association with cholestatic disease or overlap syndromes, whereas presentations dominated by isolated pruritus have been reported mainly as sporadic case reports rather than in population-based studies. Pruritus is more commonly associated with cholestatic liver diseases such as PBC and PSC, where alterations in pruritogenic mediators and bile acid handling play a central role [3]. In contrast, AIH is not classically cholestatic, making pruritus an unexpected initial manifestation that may mislead clinicians toward dermatologic or allergic conditions before hepatic pathology is considered.

Although the patient’s liver enzyme profile showed a cholestatic tilt, most notably a markedly elevated GGT with only mild ALP elevation, this pattern does not exclude AIH [1,5]. Cholestatic enzyme elevation has been described in AIH, including in cases with periportal inflammatory activity and mixed biochemical patterns [1,2]. PBC and other cholestatic disorders were excluded through a negative antimitochondrial antibody (AMA) and the absence of biliary ductal dilation on ultrasound, consistent with diagnostic guidelines [1,5]. Therefore, the biochemical profile remains compatible with inflammatory hepatocellular injury in AIH rather than a primary cholestatic process [2,5]. The presence of mild icterus with modest elevations in ALP, GGT, and bilirubin was interpreted as a secondary cholestatic component related to hepatocellular inflammation rather than evidence of a primary cholestatic liver disease.

Although pruritus is classically associated with cholestatic liver diseases, several pruritogenic pathways described in cholestasis may also provide insight into mechanisms of itch in noncholestatic inflammatory liver conditions. Key pathways include dysregulation of endogenous opioidergic signaling, activation of the lysophosphatidic acid (LPA)-autotaxin axis, and cytokine-mediated sensitization of cutaneous C-fibers [3,6]. The LPA-autotaxin pathway has been strongly implicated in cholestatic pruritus and, while not specifically studied in AIH, may help conceptualize shared mechanisms across hepatic inflammation. Furthermore, chronic pruritus guidelines emphasize that itch often arises from a combination of cholestatic, inflammatory, neuropathic, and systemic pathways, underscoring its multifactorial nature [7]. Although serum bile acids were not measured, a limitation, the predominantly hepatocellular pattern and rapid resolution of pruritus following immunosuppression suggest an inflammatory rather than cholestatic driver of symptoms.

This patient’s presentation highlights the importance of considering underlying liver disease when evaluating persistent unexplained pruritus, particularly when dermatologic causes have been excluded. The markedly elevated ALT and AST, significantly raised GGT, and the presence of high-titer ANA and ASMA strongly supported an autoimmune etiology. Viral hepatitis, Wilson disease, hemochromatosis, alpha-1 antitrypsin deficiency, and thyroid disorders were appropriately ruled out, strengthening the clinical diagnosis.

Although liver biopsy is the diagnostic gold standard for AIH and provides important information for disease confirmation and staging, it was not performed in this case because the patient declined the procedure after shared decision-making discussions [1,5]. Diagnostic confidence remained high based on the predominantly hepatocellular enzyme pattern, elevated IgG, high-titer ANA and ASMA, negative AMA result, absence of biliary abnormalities on ultrasound, and the rapid, sustained clinical and biochemical response to corticosteroid therapy. Taken together, these features fulfilled a probable/definite classification under the simplified IAIHG criteria and made alternative diagnoses, such as PBC or AIH-PBC overlap, unlikely [1,2,4,5].

Using the simplified IAIHG scoring system, the patient met the following criteria: positivity for ANA and ASMA at high titer (2 points), elevated IgG above the upper limit of normal (2 points), and exclusion of viral hepatitis and other alternative causes of liver disease (2 points). Liver histology was not available due to patient refusal (0 points). This resulted in a total simplified IAIHG score of 6, consistent with a diagnosis of probable AIH according to established thresholds. While the simplified IAIHG scoring system classifies this case as probable AIH, the diagnosis was retained based on integrated clinical, serological, and biochemical features and treatment response, consistent with accepted clinical practice and guideline recommendations [5].

Imaging findings in AIH are often nonspecific but useful for excluding biliary obstruction or other structural causes of liver injury. The absence of ductal dilation supported a noncholestatic hepatic process and made obstructive pathology or PBC less likely. Mild hepatomegaly without architectural distortion is consistent with early AIH.

Prompt initiation of corticosteroids is the cornerstone of AIH therapy, and early biochemical response is an important prognostic marker. Most patients demonstrate a significant decline in transaminase levels within weeks of starting corticosteroids [1]. Following initiation of corticosteroid therapy, the patient demonstrated a rapid biochemical response, with ALT and AST levels declining by more than 50% within two weeks and approaching near-normal values by six weeks, consistent with recognized treatment response patterns in AIH. Azathioprine remains the preferred steroid-sparing agent for long-term maintenance and was appropriately introduced following confirmation of normal TPMT activity [1,5].

This case underscores the risk of diagnostic delay in patients who present with atypical manifestations. Without timely recognition and treatment, AIH may progress to fibrosis, cirrhosis, decompensation, or hepatocellular carcinoma [1,2]. Awareness of unusual presentations, such as isolated pruritus, broadens diagnostic consideration and helps prevent progression to irreversible liver injury. This case further highlights the importance of interdisciplinary collaboration between dermatology, primary care, and hepatology in evaluating persistent pruritus unresponsive to conventional treatment.

## Conclusions

This case illustrates an atypical presentation of AIH, in which severe generalized pruritus occurred despite only mild cholestatic features and in the absence of classic hepatic symptoms. Such presentations may lead to diagnostic delay, emphasizing the importance of considering AIH in patients with persistent pruritus unresponsive to initial symptomatic therapies.

Early initiation of corticosteroid therapy resulted in rapid symptomatic improvement and biochemical remission. This case highlights the need for clinicians across specialties to recognize pruritus as a potential early manifestation of AIH. It reinforces the need for multidisciplinary awareness of atypical hepatic presentations and the value of prompt evaluation to prevent progression to advanced fibrosis or cirrhosis. Although histological confirmation was not obtained, the diagnosis was supported by integrated clinical, serological, and biochemical findings and a characteristic therapeutic response, consistent with guideline-supported diagnostic practice.
